# Extreme Diversity in the Regulation of Ndt80-Like Transcription Factors in Fungi

**DOI:** 10.1534/g3.115.021378

**Published:** 2015-10-22

**Authors:** Margaret E. Katz, Sarah Cooper

**Affiliations:** Molecular and Cellular Biology, University of New England, Armidale, NSW 2351 Australia

**Keywords:** *Aspergillus nidulans*, XprG, Ndt80, Ime2, nutrient stress

## Abstract

The *Saccharomyces cerevisiae* Ndt80 protein is the founding member of a class of p53-like transcription factors that is known as the NDT80/PhoG-like DNA-binding family. The number of *NDT80*-like genes in different fungi is highly variable and their roles, which have been examined in only a few species, include regulation of meiosis, sexual development, biofilm formation, drug resistance, virulence, the response to nutrient stress and programmed cell death. The protein kinase Ime2 regulates the single *NDT80* gene present in *S. cerevisiae*. In this study we used a genetic approach to investigate whether the *Aspergillus nidulans* Ime2 homolog, ImeB, and/or protein kinases MpkC, PhoA and PhoB regulate the two *NDT80*-like genes (*xprG* and *ndtA*) in *A. nidulans*. Disruption of *imeB*, but not *mpkC*, *phoA* or *phoB*, led to increased extracellular protease activity and a defect in mycotoxin production similar to the *xprG1* gain-of-function mutation. Quantitative RT-PCR showed that ImeB is a negative regulator of *xprG* expression and XprG is a negative regulator of *xprG* and *ndtA* expression. Thus, in contrast to Ime2, which is a positive regulator of *NDT80* in *S. cerevisiae*, ImeB is a negative regulator as in *Neurospora crassa*. However, the ability of Ndt80 to autoregulate *NDT80* is conserved in *A. nidulans* though the autoregulatory effect is negative rather than positive. Unlike *N. crassa*, a null mutation in *imeB* does not circumvent the requirement for XprG or NdtA. These results show that the regulatory activities of Ime2 and Ndt80-like proteins display an extraordinarily level of evolutionary flexibility.

The class of p53-like transcription factors that is known as the NDT80/PhoG-like DNA-binding family (http://pfam.xfam.org/family/PF05224) is found only in the unikont lineage, which includes animals, fungi and amoebozoa. The number of *NDT80*-like genes varies in different fungi and even varies within the same species/species complex (Katz *et al.* 2013; Katz and Kelly 2010). Most basidiomycetes appear to have no genes in this class whereas the Mucoromycotina fungi *Mucor circinelloides* and *Rhizopus delemar* have six and seven *NDT80*-like genes, respectively. Within the Ascomycota, the number of genes ranges from zero (*Schizosaccharomyces pombe*) to six (*Fusarium oxysporum* strain HDV247).

An analysis of all the Ndt80 homologs in a species has been completed in only three fungi, the haploid ascomycetes *Saccharomyces cerevisiae*, *Aspergillus nidulans*, and *Neurospora crassa*, which possess one, two, and three *NDT80*-like genes, respectively. In the budding yeast, *S. cerevisiae*, Ndt80 activates the transcription of more than 150 genes during the middle phase of meiosis (Chu *et al.* 1998). Ndt80 is required for completion of meiosis, which is triggered by nutrient limitation in yeast (Kupiec *et al.* 1997). Mutants lacking a functional copy of the *NDT80* gene arrest during pachytene in meiosis I at the final nutritional checkpoint (Xu *et al.* 1995). The opportunistic pathogen *Candida albicans* is a diploid ascomycete that can switch between yeast and filamentous forms. Only one of the two or three (depending on strain) *C. albicans NDT80*-like genes has been characterized. CaNdt80 is required for antifungal drug resistance, hyphal growth, biofilm formation and virulence (Chen *et al.* 2004; Nobile *et al.* 2012; Sellam *et al.* 2010).

In the filamentous fungus, *A. nidulans*, one Ndt80-like protein (XprG) is a positive regulator that controls the response of a large number of genes to carbon starvation (Katz *et al.* 2013). Extracellular protease, mycotoxin and penicillin production are regulated by XprG (Katz *et al.* 1996, 2006, 2013). In addition, XprG regulates autolysis, a process involving hyphal fragmentation, and cell death induced by carbon starvation (Katz *et al.* 2013; Krohn *et al.* 2014). A second *A. nidulans* Ndt80-like protein (NdtA) has greater sequence similarity to Ndt80 and is required for sexual reproduction. The filamentous fungus, *N. crassa*, possesses two proteins (VIB-1 and NCU04729) that are more closely related to XprG and one that is more similar to NdtA (FSD-1) (Hutchison and Glass 2010). VIB-1 is required for expression of genes involved in heterokaryon-incompatibility programmed cell death and, like XprG, is a positive regulator of extracellular protease production (Dementhon *et al.* 2006; Hutchison and Glass 2010; Xiang and Glass 2002). Both VIB-1 and FSD-1 regulate formation of female sexual structures. The *Δfsd-1* mutant is female sterile and defective in ascospore maturation. However, FSD-1 is not required for meiosis (Hutchison and Glass 2010). No phenotypic consequences have been discovered for deletion of NCU04729 (Hutchison and Glass 2010).

In *S. cerevisiae*, Ime2 is a positive regulator of Ndt80. Activation of *NDT80* gene expression involves phosphorylation of the Sum1 repressor, which is bound to the *NDT80* promotor, by Ime2 (reviewed in Winter 2012). There is also evidence that Ndt80 requires post-translational activation and that Ime2 plays a role in this step (Sopko *et al.* 2002; Benjamin *et al.* 2003). However, it is still not clear whether it is Ime2-dependent phosphorylation that is required for Ndt80 activity (Shubassi *et al.* 2003; Sopko and Stuart 2004; Wang *et al.* 2011). The regulation of Ndt80-like proteins by Ime2 homologs has been studied in only one filamentous fungus, *N. crassa*. In contrast to *S. cerevisiae*, *N. crassa* IME-2 is a negative regulator of *vib-1* expression (Hutchison *et al.* 2012). Mutations in *ime-2* have no effect on *fsd-1* expression (Hutchison and Glass 2010) and no investigations into NCU04729 regulation have been reported. VIB-1 is phosphorylated at a site that matches the Ime2 consensus phosphorylation site. However, amino acid substitutions that were predicted to be phospho-null or phospho-mimetic had no effect on VIB-1-mediated programmed cell death (Hutchison *et al.* 2012).

In this study, we show that some aspects of the *A. nidulans* ImeB/XprG/NdtA regulatory pathway are similar to the *N. crassa* IME-2/VIB-1/FSD-1 pathway but others are not. Like IME-2, ImeB is a negative regulator of *xprG* expression. However, in *N. crassa*, *ime-2* gene disruption suppresses the defects in extracellular protease production and heterokaryon-incompatibility induced cell death associated with the Δ*vib-1* mutation and the defect in female sexual development associated with the Δ*fsd-1*mutation (Hutchison *et al.* 2012; Hutchison and Glass 2010). In contrast, in *A. nidulans* the requirement for XprG or NdtA is not circumvented by null mutations in *imeB*. We also show that XprG is a negative regulator of *ndtA* and *xprG* expression, though genetic evidence and transcriptional profiling indicate that XprG is usually a transcriptional activator. Thus, the ability of Ndt80-like proteins to regulate the transcription of *NDT80*-like genes is conserved in *S. cerevisiae* and *A. nidulans* even though in yeast the autoregulatory effect is positive whereas in *A. nidulans* it is negative. These results, coupled with the extreme variability in the number of *NDT80*-like genes, show that the regulatory activities of Ime2 and Ndt80-like proteins display an extraordinarily level of evolutionary flexibility.

## Materials and Methods

### *Aspergillus* strains and growth tests

The *A. nidulans* strains used in this study are listed in Table 1. The genetic techniques used to construct the strains listed in Table 1 have been described (Clutterbuck 1974). Growth tests were performed at 37° using *Aspergillus* minimal medium (Cove 1966). In media containing 1% glucose as a carbon source, nitrogen sources were added at a final concentration of 10 mM with the exception of skim milk or bovine serum albumin (BSA), which were used at 1%. In media that contained 1% skim milk or 1% BSA as a carbon source, 10 mM ammonium chloride was used as a nitrogen source. For media that contained skim milk, sodium deoxycholate (0.08%) was used to induce compact colony formation. Sexual development was initiated by growth on solid minimal medium containing sodium nitrate, proline, or alanine as a nitrogen source. After 3 days, air was excluded and the plates were incubated for a further 7–14 days before scoring and image capture using a Leica MZ6 stereomicroscope and Leica IC80 HD digital camera.

**Table 1:** List of *Aspergillus nidulans* strains used in this study

Strain	Genotype*^a^*	Source/Reference
A1313	*pyrG89*; *wA3*; *argB2*; *ΔnkuA*::*argB pyroA4*; *phoBΔ* (*phoB*::*pyrG^Af^*); *fwA1 chaA1 sE15 nirA14*	FGSC
A1338	*pyrG89*; *wA3*; *argB2*; *mpkCΔ* (*mpkC*::*pyrG^Af^*); *ΔnkuA*::*argB pyroA4*; *fwA1 chaA1 sE15 nirA14*	FGSC
A1357	*imeBΔ* (*imeB*::*pyrG^Af^*); *pyrG89*; *wA3*; *argB2*; *ΔnkuA*::*argB pyroA4*; *fwA1 chaA1 sE15 nirA14*	FGSC
MH2	*biA1*; *niiA4*	(Sandeman and Hynes 1989)
MH97	*pabaA1 yA1 acuE215*	(Sandeman and Hynes 1989)
MH11036	*pyroA4 ΔnkuA*::*argB*; *riboB2*	(Nayak *et al.* 2006)
MK85	*biA1*; *xprG1*; *niiA4*	(Katz *et al.* 2000)
MK414	*pabaA1 yA2*; *argB2*; *xprGΔ* (*xprG*::*argB*)	(Katz *et al.* 2006)
MK422	*biA1*; *xprGΔ*(*xprG*::*argB*)	(Katz *et al.* 2013)
MK481	*ndtAΔ* (*ndtA*::*pyroA^Af^*); *pyroA4 nkuA*::*argB*; *riboB2*	(Katz *et al.* 2013)
MK489	*hxkCΔ* (*hxkC*::*argB*); *niiA4*	This study
MK490	*biA1 acuE215*; *hxkDΔ3* (*hxkD*::*argB*); *niiA4 riboB2*	This study
MK505	*ndtAΔ* (*ndtA*::*pyroA^Af^*); *pyroA4 nkuA*::*argB*; *prnΔ309 xprG2*; *niiA4*	(Katz *et al.* 2013)
MK552	*phoAΔ* (*phoA*::*pyroA^Af^*); *pyroA4 ΔnkuA*::*argB*; *riboB2*	This study
MK562	*biA ;veA^+^*	(Katz *et al.* 2015)
MK577	*pabaA1*; *phoAΔ* (*phoA*::*pyroA^Af^*); *pyroA4 ΔnkuA*::*argB*; *xprG1*	This study
MK578	*phoAΔ* (*phoA*::*pyroA^Af^*); *pyroA4 ΔnkuA*::*argB*; *xprGΔ* (*xprG*::*argB*)	This study
MK582	*imeBΔ* (*imeB*::*pyrG^Af^*) *pabaA1 yA1 acuE215*; *ΔnkuA*::*argB*; *xprG1*; *fwA1*	This study
MK598	*imeBΔ* (*imeB*::*pyrG^Af^*) *biA1*; *argB2*; *ΔnkuA*::*argB*; *niiA4 chA1*	This study
MK601	*imeBΔ* (*imeB*::*pyrG^Af^*) *biA1*; *argB2*; *xprGΔ* (*xprG*::*argB*)	This study
MK604	*argB2*; *pyroA4 ΔnkuA*::*argB*; *xprGΔ* (*xprG*::*argB*) *phoBΔ* (*phoB*::*pyrG^Af^*)	This study
MK606	*xprG1 phoBΔ* (*phoB*::*pyrG^Af^*)	This study
MK607	*phoBΔ* (*phoB*::*pyrG^Af^*)	This study
MK608	*pabaA1*; *mpkCΔ* (*mpkC*::*pyrG^Af^*) *argB2*; *ΔnkuA*::*argB*; *xprGΔ* (*xprG*::*argB*)	This study
MK609	*mpkCΔ* (*mpkC*::*pyrG^Af^*) *argB2*; *pyroA4 ΔnkuA*::*argB*; *nirA14*	This study
MK612	*mpkCΔ* (*mpkC*::*pyrG^Af^*); *xprG1*	This study
MK643	*imeBΔ* (*imeB*::*pyrG^Af^*) *biA1*; *argB2*; *xprGΔ* (*xprG*::*argB*); *veA^+^*	This study
MK645	*imeBΔ* (*imeB*::*pyrG^Af^*) *biA1*; *veA^+^*	This study
MK647	*imeBΔ* (*imeB*::*pyrG^Af^*) *ndtAΔ (ndtA*::*pyroA^Af^) biA1*; *pyroA4 ΔnkuA*::*argB*; *riboB2 nirA14*	This study
MK649	*imeBΔ* (*imeB*::*pyrG^Af^*) *ndtAΔ (ndtA*::*pyroA^Af^) biA1*; *argB2*; *pyroA4 ΔnkuA*::*argB*; *xprG2 niiA4*	This study

a The gene symbols are described in the *Aspergillus* Genome Database (http://www.aspgd.org/). FGSC, Fungal Genetics Stock Center

### Protein kinase mutants

The *A. nidulans* genome contains two genes that have been designated *phoA*, AN8261, which encodes the cyclin-dependent protein kinase, and AN4055, which encodes a putative acid phosphatase. The entire AN8261 coding region (chromosome II coordinates 1,314,068-1,315,312; AspGD) was replaced with the *Aspergillus fumigatus pyroA* gene using a strategy similar to the one described in Nayak *et al.* (2006). Gene disruption was confirmed by PCR using the primers listed in Supporting Information, Table S1, and Southern blot analysis.

The A1313, A1338 and A1357 strains, which carry disruptions of the *A. nidulans phoB* (AN1867), *mpkC* (AN4668) and *imeB* (AN6243) genes, respectively, were obtained from the Fungal Genetics Stock Center (McCluskey *et al.* 2010). These strains were crossed to obtain kinase mutants that did not carry the *sE15* mutation, which requires supplementation with methionine and, as a consequence, interferes with growth tests. The presence of *mpkC*:: *pyrG^Af^* (*mpkCΔ*) and *phoB*:: *pyroA^Af^* (*phoBΔ*) in segregants was confirmed by PCR using the primers listed in Table S1 as these mutants could not be scored based on growth morphology. The *imeB*::*pyrG^Af^* (*imeBΔ*) mutation results in reduced growth and compact colony morphology.

### Assay for extracellular protease activity, sterigmatocystin, and autolysis

To measure production of extracellular proteases in response to carbon or nitrogen starvation, mycelia were grown in minimal medium containing glucose and ammonium tartrate, and then transferred to minimal medium containing no carbon source for 16 hr or no nitrogen source for 4 hr. Filtered culture medium was used in protease enzyme assays as described previously (Katz *et al.* 1996).

To measure production of sterigmatocystin (ST) in response to carbon starvation, mycelia were grown for 24 hr in minimal medium containing glucose and then transferred to minimal medium containing glucose for 24 hr or no carbon source for 24 or 48 hr. A volume of culture filtrate corresponding to 10 mg of mycelial dry weight was lyophilized and then resuspended in 1 ml of water. The ST was extracted with 1 ml of chloroform and then repeated with 0.5 ml of chloroform. After evaporation of the chloroform, the sample was resuspended in 25 µl of chloroform. ST was detected using a method described previously (Keller *et al.* 1994). A 5-µl sample of each extract was applied to aluminum-backed, silica thin layer chromatography sheets (Merck, Darmstadt, Germany) and separated using a mixture of benzene and glacial acetic acid (95:5). After drying, the plate was sprayed with 15% AlCl_3_ dissolved in 95% ethanol, baked at 65° for 15 min, and photographed under 365 nm UV illumination. ST (Sigma, St. Louis, MO) was used as a standard.

The progress of autolysis in submerged cultures following nutrient depletion was monitored as described previously (Katz *et al.* 2013). For each assay, six flasks containing 50 ml of minimal medium, 10 mM ammonium tartrate and vitamin supplements were each inoculated with 3 × 10^8^ conidia and placed on an orbital shaker. Flasks were removed at 24 or 48 hr intervals, photographed and the weight of dried mycelium recorded. Each strain was assayed three times.

### RNA extraction and qRT-PCR

Total RNA was extracted from mycelia transferred to medium containing glucose or no carbon source for 16 hr as described previously (Reinert *et al.* 1981) and treated with the Ambion Turbo DNA-free Kit (Invitrogen, AM1907, Carlsbad, CA) prior to quantification in a SpectraMax M2e Microplate Reader (Molecular Devices, M2E, Sunnyvale, CA). The primers used in qRT-PCR experiments were designed using the Primer3 program (http://frodo.wi.mit.edu/primer3/) and are listed in Table S1. Each primer pair was first tested with serial dilutions of RNA to determine the linear range of the qRT-PCR assays using the SuperScript III Platinum SYBR Green One-Step qRT-PCR Kits (Invitrogen, 11736). The experiments were performed using a Corbett CAS1200 liquid handling robot and Corbett Rotor-Gene 3000 real-time thermal cycler (QIAGEN, RG3000, Hilden, Germany). In the assays to determine relative transcript levels, 1 ng of total RNA was added to each reaction. A minimum of three independent RNA preparations were assayed.

### Data availability Strains available upon request.

## Results

### Similarity of ImeB, MpkC, PhoA and PhoB to Ime2

The *S. cerevisiae* Ime2 protein kinase regulates the transcription and activity of the Ndt80 transcription factor. In contrast to *S. cerevisiae*, which possesses a single gene encoding an Ndt80-like transcription factor, *A. nidulans* possesses two genes encoding XprG and NdtA (Katz *et al.* 2006, 2013). Ndt80 shows greater similarity to NdtA (17.1% identity, E value 4.0e–09) than XprG (12.4% identity, E value 1.6). Like Ndt80, NdtA is required for sexual reproduction. We therefore considered the possibility that NdtA might be regulated by the *A. nidulans* homolog of Ime2 while XprG might be regulated by a different protein kinase. The four protein kinases that showed the greatest similarity to *S. cerevisiae* Ime2 are listed in Table 2. ImeB, at 781 amino acids in length, is similar in size to *S. cerevisiae* Ime2, which is 645 amino acids while MpkC, PhoB and PhoA are smaller. However, all four *A. nidulans* protein kinases show a high degree of similarity to the N-terminal half of Ime2 (Figure S1). The phenotype of the *phoA1* deletion strain constructed by Bussink and Osmani (1998) showed some similarities with the *xprG1* gain-of-function mutant, including an altered response to phosphate limitation and increased secretion of pigment (Katz *et al.* 2006). A mutant in which the entire *phoA* coding region was removed was constructed and deletion mutants for *imeB*, *mpkC* and *phoB* (De Souza *et al.* 2013) were obtained from the Fungal Genetics Stock Center (McCluskey *et al.* 2010).

**Table 2:** *Aspergillus nidulans* protein kinases showing highest similarity to *Saccharomyces cerevisiae* Ime2

Protein Kinase	No. of Identical Amino Acids	No. of Identical + Similar Amino Acids	Length of Protein (Amino Acids)	% Identity	E Value	Phenotype of Deletion Mutants
ImeB AN6243	201	445	736	40.6	1.0e–77	Slow growth, abnormal sexual development, reduced production of sterigmatocystin (Bayram *et al.* 2009), cold sensitivity (De Souza *et al.* 2013)
MpkC AN4668	135	305	415	35.6	2.0e–29	None noted (De Souza *et al.* 2013; Furukawa *et al.* 2005)
PhoA AN8261	115	255	366	29.2	8.0e–28	Pigment secretion increased, conidiation decreased and sexual development increased in response to phosphate limitation (Bussink and Osmani 1998), lethal in combination with Δ*phoB* (Dou *et al.* 2003), NaCl sensitivity, marginal hydroxyurea sensitivity (De Souza *et al.* 2013)
PhoB AN1867	103	224	313	30.0	1.0e–27	Lethal in combination with Δ*phoA* (Dou *et al.* 2003)

**Figure 1:**
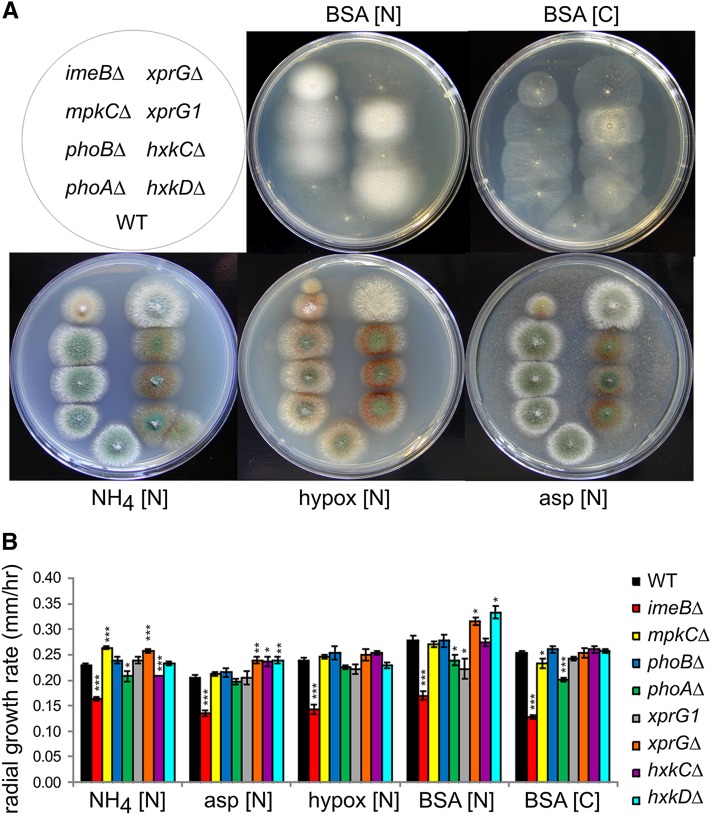
Phenotypic comparison of protein kinase mutants with strains carrying mutations in the *xprG*, *hxkC* and *hxkD* genes. Colony morphology (A) and radial growth rate (B) on media containing bovine serum albumin (BSA), ammonium tartrate (NH_4_), hypoxanthine (hypox) or aspartic acid (asp) as a nitrogen source [N] or BSA as a carbon source [C]. The full genotypes for the wild type (WT) (MH2), *imeBΔ* (MK598), *mpkC*Δ (MK609), *phoBΔ* (607), *phoAΔ* (MK552), *xprGΔ* (MK422), *xprG1* (MK85), *hxkCΔ* (MK489) and *hxkDΔ* (MK490) strains are given in Table 1. Radial growth rate was measured between 20 and 44 hr after inoculation. For each strain, the average growth rate and standard error for three colonies grown on three separate plates are shown. An unpaired *t*-test was used to analyze the data. Values that differed significantly from the value for the WT strain are indicated with asterisks (* *P* ≤ 0.05, ** *P* ≤ 0.01, *** *P* ≤ 0.001).

In *A. nidulans*, colony morphology is determined by the radial growth rate, density of hyphae, conidiation, and production of pigments. As reported previously, we found that the *imeBΔ* mutation results in reduced radial growth rate and compact colony morphology (Figure 1, Bayram *et al.* 2009; De Souza *et al.* 2013), and conidiation in the *phoAΔ* mutant is reduced (Bussink and Osmani 1998). The colony morphology of the *mpkCΔ* and *phoBΔ* mutants was indistinguishable from control strains on *Aspergillus* complete medium. The *hxkC* and *hxkD* genes encode noncatalytic, hexokinase-like proteins (Bernardo *et al.* 2007; Katz *et al.* 2000). Genetic evidence suggests that HxkC and HxkD are negative regulators of XprG. The *hxkCΔ* and *hxkDΔ* loss-of-function mutations and *xprG1* gain-of-function mutations are associated with sparse hyphae and production of brown pigment on media containing nitrogen sources other than protein (Bernardo *et al.* 2007; Katz *et al.* 2006). No such growth defects were observed in the *imeBΔ*, *mpkCΔ*, *phoAΔ*, and *phoBΔ* mutants but the *imeBΔ* mutant displayed much denser hyphae due to stronger growth on media containing protein (BSA) as a nitrogen source, similar to the *xprG1* and *hxkDΔ* mutants (Figure 1A).

### Extracellular protease production is elevated in the *imeBΔ* mutant

**Figure 2:**
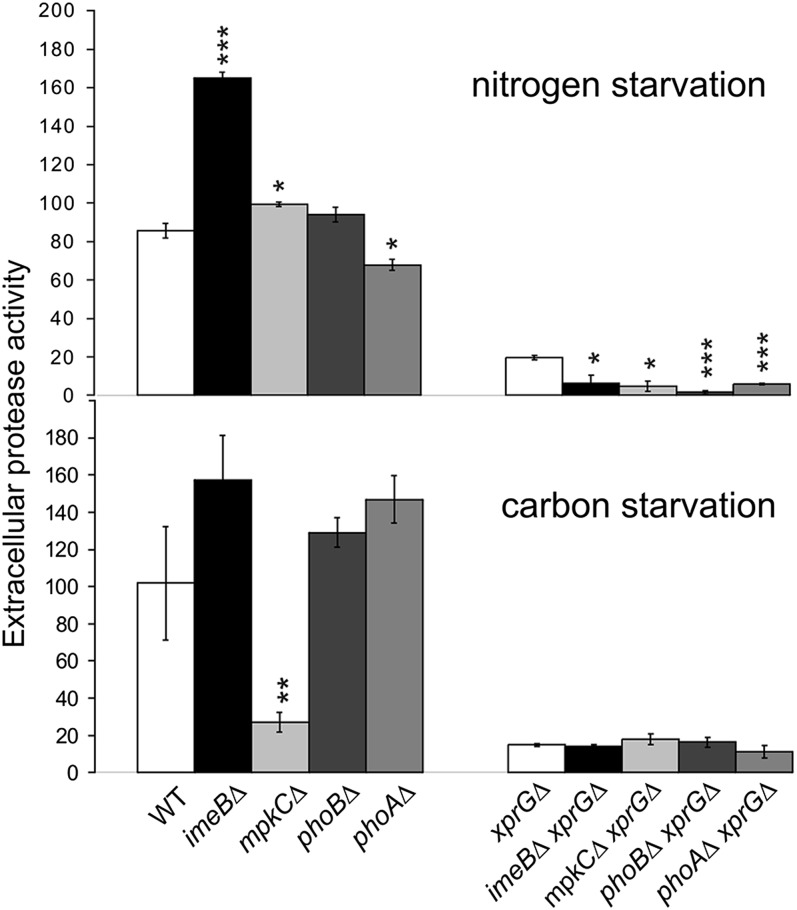
Extracellular protease enzyme activity in the protein kinase mutants. The effect of 4 hr of nitrogen starvation or 16 hr carbon starvation on extracellular protease activity was measured in protease enzyme assays using azocasein as a substrate. Protease activity was calculated as total absorbance units per gram (dry weight) of mycelium and is expressed in arbitrary units. The results are the average for a minimum of three cultures and standard errors are shown. An unpaired *t*-test was used to analyze the data. Values that differed significantly from the value for the WT strain (for the single mutants) or the *xprGΔ* strain (for the double mutants) are indicated with asterisks (* *P* ≤ 0.05, ** *P* ≤ 0.01, *** *P* ≤ 0.001). The full genotypes for the WT (MH2), *imeBΔ* (MK598), *mpkC*Δ (MK609), *phoAΔ* (MK552), *phoBΔ* (607), *imeBΔ xprGΔ* (MK601), *mpkC*Δ *xprGΔ* (MK608), *phoAΔ xprGΔ* (MK578), *phoBΔ xprGΔ* (604), and *xprGΔ* (MK422) strains are given in Table 1.

Mutations in *xprG* but not *ndtA* alter extracellular protease production (Katz *et al.* 2013). The *xprG1* gain-of-function mutation increases extracellular protease production in response to carbon and nitrogen starvation whereas in loss-of-function mutants (*e.g.*, *xprGΔ*), protease production is reduced (Katz *et al.* 1996, 2006, 2008). We examined the effect of the *imeBΔ*, *mpkCΔ*, *phoAΔ*, and *phoBΔ* mutations on extracellular protease levels and the interaction of the kinase mutations with the two types of *xprG* mutation using skim milk agar and protease enzymes assays (Figure S2 and Figure 2). These assays showed that the *imeB* mutation leads to an increase in extracellular protease levels in response to nitrogen limitation and suggested that ImeB might be a negative regulator of protease production. However, protease enzyme activity levels were very low in the *imeBΔ xprGΔ* double mutant. This result was unexpected. In *N. crassa*, protease deficiency due to mutations in the *xprG* homolog, *vib-1*, is suppressed by the *Δime-2* mutation (Hutchison *et al.* 2012). Previous studies on the *A. nidulans imeB* gene have reported that some *imeBΔ* mutant phenotypes are expressed only in a *veA^+^* genetic background (Bayram *et al.* 2009). Most laboratory strains of *A. nidulans* carry the *veA1* point mutation, which allows asexual spore production in the absence of light (Kim *et al.* 2002). An *imeBΔ xprGΔ veA^+^* strain was constructed to test whether *imeBΔ* was able to restore protease production in an *xprGΔ* mutant with a wild-type version of the VeA light sensor. When tested on solid medium containing skim milk, the phenotype of the *imeBΔ xprGΔ veA^+^* and *imeBΔ xprGΔ veA1* strains were indistinguishable—both were protease-deficient (Figure S2). Thus, it is likely that that the increase in protease production in the *imeBΔ* mutant is mediated by XprG.

The *phoBΔ* deletion mutation did not alter protease production but, during nitrogen starvation, extracellular protease levels were significantly reduced in the *phoAΔ* mutant (Figure 2). When tested on skim milk agar, no changes in protease levels were detect in the *mpkCΔ* mutant (Figure S2), but in the enzyme assay protease activity in response to carbon starvation was much lower than in the wild-type strain (Figure 2). Discrepancies between the milk-clearing and protease enzyme assays could be due to the difference in growth conditions used in the two assays. In the enzyme assays no carbon/nitrogen source is provided whereas both milk and low molecular weight carbon/nitrogen sources are present in the skim milk agar.

### Protein kinases do not play a role in autolysis but ImeB regulates mycotoxin synthesis

**Figure 3:**
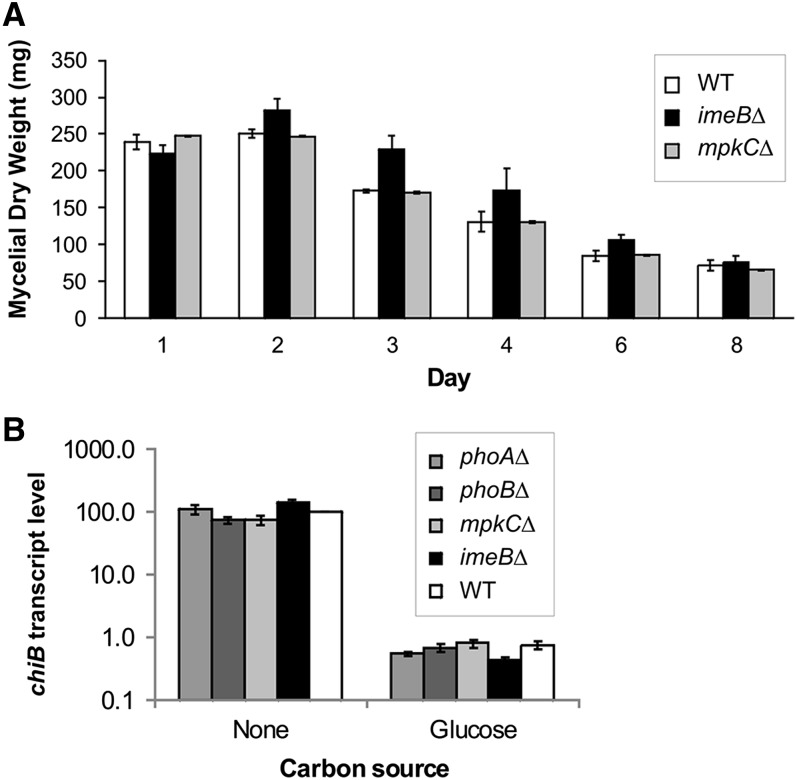
Effect of protein kinase mutants on autolysis. (A) Loss of mycelial mass was monitored for 8 days in submerged cultures inoculated with 3 × 10^8^ conidia. The average for the three experiments and standard errors are shown. The mycelial dry weight of the two mutants did not differ from the mass of the WT strain at each time point when the data were analyzed using an unpaired *t*-test. (B) Levels of the *chiB* transcript relative to *actA* mRNA levels. The *chiB* encoded chitinase is a marker of autolysis. The results are the average for three independent RNA preparations, each of which was assayed in duplicate. Transcript levels and standard errors, relative to the levels in the WT control during carbon starvation, are shown. Note the log scale on the x-axis. The data were analyzed using ANOVA after log*_e_* transformation. The 95% confidence intervals for all four mutants overlapped with the 95% confidence intervals for the WT strain. The strain numbers for the mutants is given in the legend of Figure 2, and the full genotypes are listed in Table 1.

Autolysis, which occurs in stationary submerged cultures after carbon source depletion, is associated with an increase in extracellular protease and chitinase activity, loss of mycelial mass, accumulation of dark pigment, hyphal fragmentation and disintegration (Emri *et al.* 2004, 2005). Analysis of extracellular protease production indicated that ImeB could be a negative regulator of XprG and, as such, the *imeBΔ* mutant would be predicted to cause accelerated autolysis, similar to the *xprG1* mutant. However, there was no evidence that autolysis occurred more rapidly in the *imeBΔ* mutant (Figure 3A). Autolysis was also examined in the *mpkCΔ* mutant, which showed low levels of extracellular protease in response to carbon starvation in protease enzyme assays. The loss of mycelial mass that occurs as a result of autolysis was not delayed in the *mpkCΔ* mutant as it is in the absence of XprG.

Carbon starvation–induced autolysis is accompanied by increased expression of the chitinase gene, *chiB* (Yamazaki *et al.* 2007), therefore this gene can be used as a reporter of autolysis. Unlike the *xprGΔ* mutant, which has less than 20% of wild-type levels *chiB* transcript in carbon-starved mycelia, and the *xprG1* mutant, which has elevated levels of *chiB* mRNA in nutrient-sufficient medium (Katz *et al.* 2015), none of the kinase mutants showed altered *chiB* transcript levels (Figure 3B).

**Figure 4:**
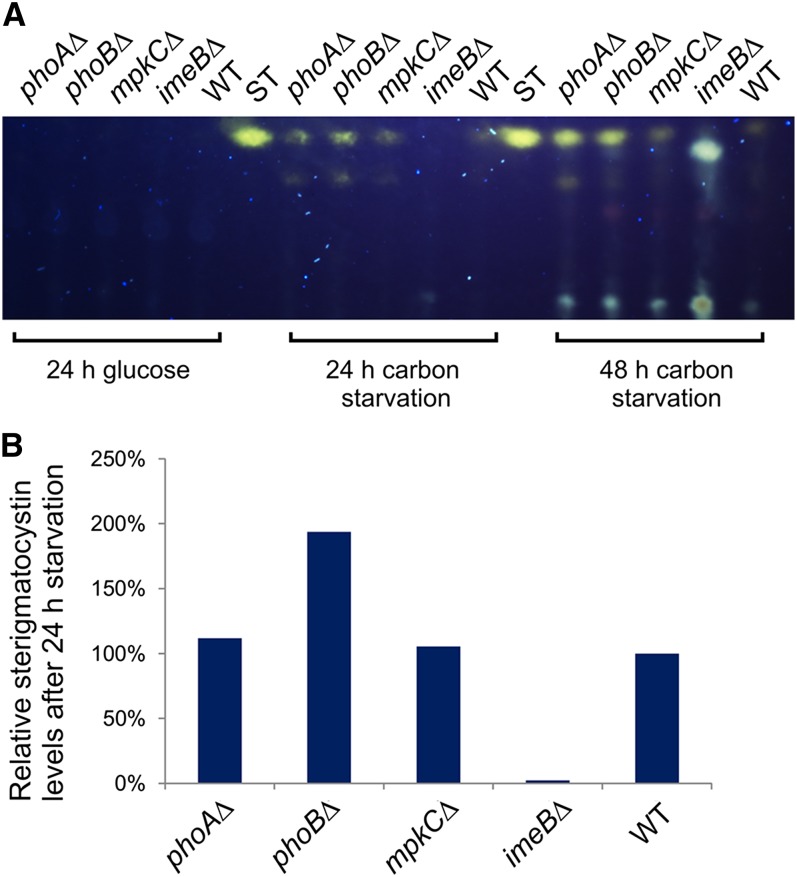
Sterigmatocystin levels in the protein kinase mutants. (A) Sterigmatocystin extracted from culture medium containing glucose or no carbon source was analyzed using thin layer chromatography. Sterigmatocystin fluoresces yellow under ultraviolet light after treatment with AlCl_3_. Each sample was extracted from culture filtrate corresponding to 2 mg of mycelia (dry weight). In the 48-hr extract from the *imeBΔ* mutant, there is a bright blue band below the faint yellow sterigmatocystin band. Sterigmatocystin (ST) (Sigma) was applied as a standard. (B) Sterigmatocystin levels, relative to the WT strain, after 24 hr of carbon starvation. Sterigmatocystin levels were quantified using ImageJ software (Rasband 1997–2014). The strain numbers for the mutants are given in the legend of Figure 2 and the full genotypes are listed in Table 1.

It has previously been reported that ImeB is required for production of the mycotoxin, sterigmatocystin, which is a precursor of aflatoxin (Bayram *et al.* 2009). We examined production of sterigmatocystin in response to carbon starvation in the kinase mutants (Figure 4). After 24 hr in carbon-free medium, sterigmatocystin was detected in the *phoAΔ*, *phoBΔ* and *mpkCΔ* mutants and control strain but not in the *imeBΔ* mutant. After 48 hr, a faint band corresponding in position to sterigmatocystin was visible in extracts from the *imeBΔ* mutant. These results are consistent with the findings of Bayram *et al.* (2009), in spite of the differences in culture conditions and genetic background, and show that ImeB-mediated regulation of mycotoxin production does not depend on the presence of the wild-type *veA^+^* allele.

### XprG is a negative regulator and NdtA is a positive regulator of sexual development

**Figure 5:**
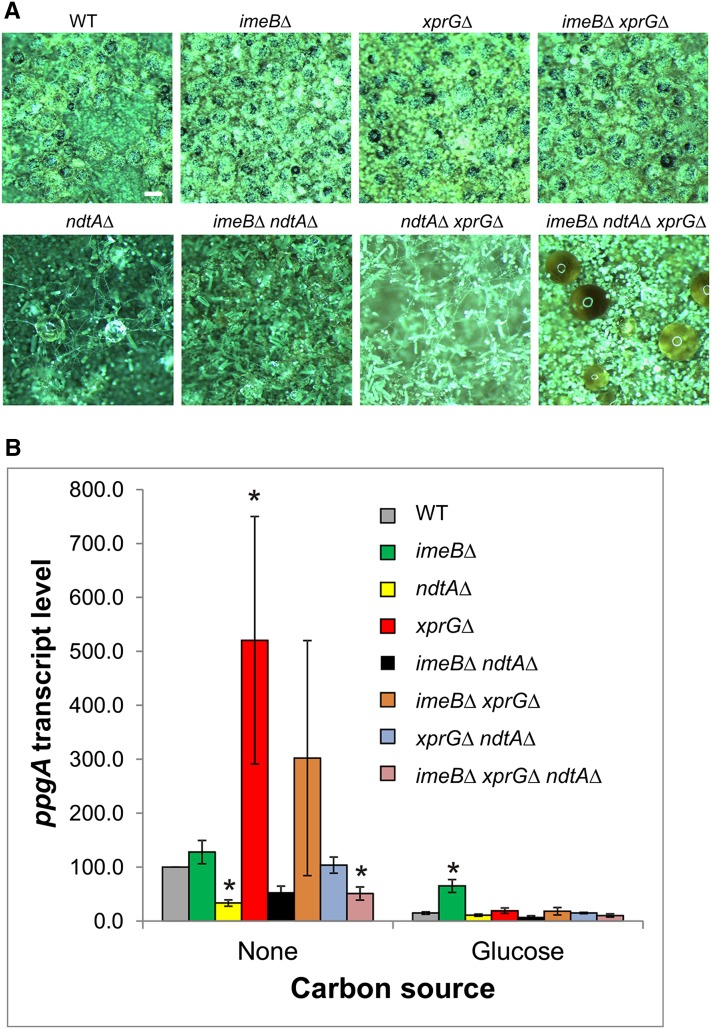
Sexual reproduction in *imeBΔ*, *ndtAΔ* and *xprGΔ* mutants. (A) Formation of sexual fruiting bodies (cleistothecia). Mature cleistothecia, which are shiny opaque black spheres, are visible in the pictures in the top row. Each picture shows a 2 mm × 2 mm section of a colony. A white scale bar (200 µm) is shown in the WT picture. The white circles on the clear spherical structures in the second row of pictures are a reflection of the stereomicroscope lights. (B) Levels of the *ppgA* transcript, which encodes a putative sex pheromone, relative to *actA* mRNA levels. The results are the average for a minimum of three independent RNA preparations, each of which was assayed in duplicate. Transcript levels and standard errors, relative to the levels in the WT control during carbon starvation, are shown. The data were analyzed using ANOVA after log*_e_* transformation. The values marked with an asterisk are outside the 95% confidence intervals for the WT strain. The full genotypes of the WT (MH2), *imeBΔ* (MK598), *ndtAΔ* (MK481), *xprGΔ* (MK422), *imeBΔ ndtAΔ* (MK647), *imeBΔ xprGΔ* (MK601), *xprGΔ ndtAΔ* (MK505), and *imeBΔ xprGΔ ndtAΔ* (MK649) strains are given in Table 1.

The genetic interactions between the *imeBΔ* and *ndtAΔ* mutations were examined to test whether ImeB is involved in regulating NdtA, the second member of the Ndt80/PhoG class of transcription factors. It has previously been shown that NdtA is required for development of sexual fruiting bodies (cleistothecia) in *A. nidulans* (Katz *et al.* 2013). As *A. nidulans* is self-fertile, the ability of *imeBΔ*, *xprGΔ* and *ndtAΔ* single, double and triple mutants to complete sexual development on media containing a variety of nitrogen sources was examined. The *imeBΔ*, *xprGΔ*, *xprG1* and *imeBΔ xprGΔ* mutants formed large numbers of cleistothecia in the selfing assays but no cleistothecia were detected in the *ndtAΔ*, *imeBΔ ndtAΔ*, *ndtAΔ xprGΔ* or *imeBΔ ndtAΔ xprGΔ* mutants (Figure 5A and data not shown). Thus, neither *imeBΔ* nor *xprGΔ* are able to suppress the *ndtAΔ* developmental defect. Large clear spheres were observed in the selfing plates of the *ndtAΔ* mutants. Similar macroscopic aggregates of Hulle cells, which normally surround the cleistothecium, were seen in strains lacking the *MAT1* or *MAT2* mating type genes (Paoletti *et al.* 2007). In the *imeBΔ ndtAΔ xprGΔ* triple mutant, these aggregates were very large and dark in coloring (Figure 5A).

The *ppgA* gene, which encodes a putative sex pheromone similar to *S. cerevisiae* α-factor, is upregulated during sexual development (Paoletti *et al.* 2007). Expression of *ppgA* and other genes involved in sexual development is increased in the *xprGΔ* mutant (Katz *et al.* 2013). To further investigate the role of ImeB in sexual reproduction, *ppgA* transcript levels were examined in *imeBΔ*, *xprGΔ* and *ndtAΔ* mutants using qRT-PCR (Figure 5B). The results show that *ppgA* expression is increased during carbon starvation and is dependent on NdtA. In the *xprGΔ* single mutant but not the *xprGΔ ndtAΔ* double mutant, carbon-starvation-induced *ppgA* expression is greatly increased. In nutrient-sufficient conditions, the *imeBΔ* mutation leads to increased *ppgA* transcript levels. However, there was no evidence that ImeB repressed *ppgA* expression during carbon starvation.

### Regulation of *ndtA* and *xprG* expression

**Figure 6:**
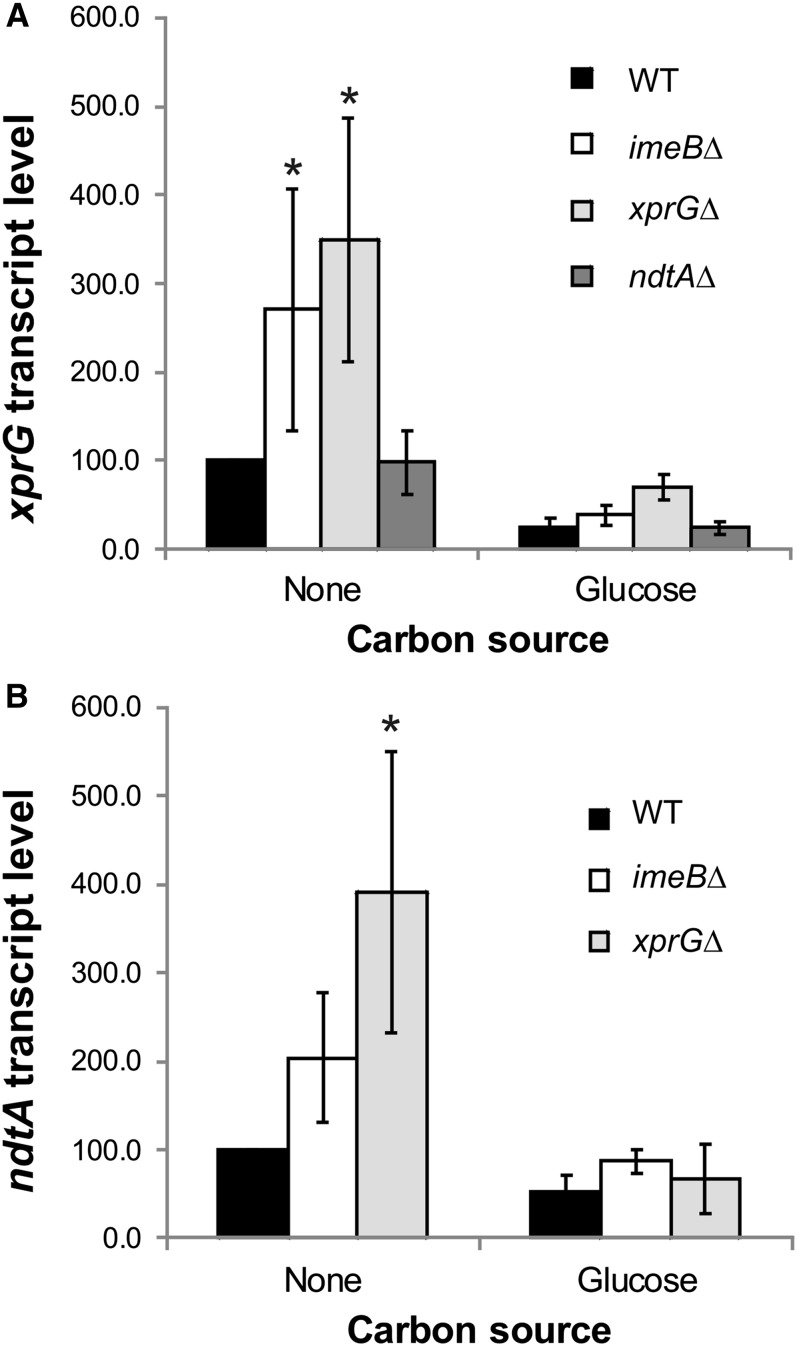
Effect of the *imeBΔ*, *ndtAΔ* and *xprGΔ* mutation on *xprG* (A) and *ndtA* (B) transcript levels relative to *actA* mRNA levels. The results are the average for three to six independent RNA preparations. Transcript levels and standard errors, relative to the levels in the WT control during carbon starvation, are shown. The data were analyzed using ANOVA after log*_e_* transformation. The values marked with an asterisk are outside the 95% confidence intervals for the WT strain. The full genotypes of the WT (MH2), *imeBΔ* (MK598), *ndtAΔ* (MK481), and *xprGΔ* (MK422) strains are given in Table 1.

**Figure 7:**
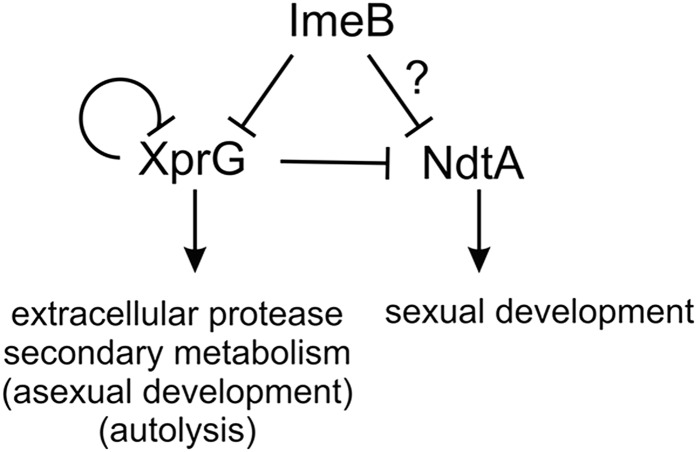
Model for transcriptional control of the ImeB/XprG/NdtA regulatory pathway. ImeB is a negative regulator of *xprG* transcription and may also regulate *ndtA* expression. XprG is a negative regulator of *ndtA* transcript levels and also has a negative autoregulatory function. XprG plays a major role in the response to carbon starvation, including activation of genes encoding extracellular proteases, secondary metabolism (including the sterigmatocystin biosynthetic pathway), genes induced during autolysis, and genes that are upregulated during asexual development while NdtA is required for sexual development (Katz *et al.* 2013). There is no evidence that ImeB modulates *xprG* expression during autolysis or asexual development.

The elevated levels of the *ppgA* transcript observed in the *xprGΔ* mutant indicated that XprG might regulate *ndtA* expression or NdtA activity, whereas the XprG-dependent increase in extracellular protease activity seen in the *imeB* mutant suggested that ImeB could be involved in the regulation of XprG. We tested these hypotheses by measuring *xprG* and *ndtA* transcript levels (Figure 6). The binding sites for the primers used to measure *xprG* transcript levels are still present in the *xprGΔ* deletion mutation, which removes codons 248–344. The relative expression of both the *xprG* and *ndtA* genes was higher in carbon-starved mycelia than in mycelia that were not subjected to nutrient stress. In the *imeBΔ* mutant, *xprG* transcript levels were much higher than in the control strain and *ndtAΔ* mutant, but only in response to carbon limitation. Carbon-starvation-induced expression of *ndtA* was greatly increased in the *xprGΔ* mutant and to a much lesser extent (which was not outside the 95% confidence interval for the control strain) in the *imeBΔ* mutant. These results are consistent with a model in which ImeB is a negative regulator of *xprG* expression and XprG is a negative regulator of *ndtA* expression (Figure 7). NdtA does not appear to regulate *xprG* expression. In the *xprGΔ* mutant, carbon-starvation-induced *xprG* transcript levels are greatly increased, indicating that XprG has an autoregulatory function.

## Discussion

The effect of the *imeBΔ* mutation in *A. nidulans* differs from similar mutations in *S. cerevisiae* and *N. crassa*. Ime2 is positive regulator of *NDT80* expression in *S. cerevisiae* (Winter 2012). In contrast, *A. nidulans* ImeB and *N. crassa* IME-2 are negative regulators of *xprG* and *vib-1* expression, respectively (Hutchison *et al.* 2012). Mutations in the *N. crassa vib-1* gene have many phenotypic effects that are similar to loss-of-function mutations in the *A. nidulans xprG* gene. Both mutations affect programmed cell death, extracellular protease production and conidial pigmentation (Dementhon *et al.* 2006; Katz *et al.* 1996, 2006, 2013; Xiang and Glass 2002). The *N. crassa Δime-2* mutation suppresses the *Δvib-1* defect in heterokaryon-incompatibility induced programmed cell death and extracellular protease production in response to nitrogen starvation but wild-type conidial pigmentation is not restored (Hutchison *et al.* 2012). The *Δime-2* mutation partially restores extracellular protease production even in a strain lacking all three *NDT80*-like genes (*vib-1*, *fsd-1*, and NCU4729) (Hutchison *et al.* 2012). The *Δfsd-1* and *Δvib-1* mutants are both defective in female sexual development. The *Δime-2* mutation suppresses this defect in the *Δfsd-1* mutant but not in the *Δvib-1* mutant. The interaction between *IME2*- and *NDT80* -like genes differs in *A. nidulans*. In contrast to the *N. crassa Δime-2* mutation, which suppresses most *Δvib-1* and *Δfsd-1* defects, the *A. nidulans imeBΔ* mutation does not suppress the *xprGΔ* defect in extracellular protease production or the *ndtAΔ* defect in sexual reproduction. As the *N. crassa Δime-2* mutation restores extracellular protease production in a strain lacking all three *NDT80*-like genes, IME-2 must regulate a parallel signaling pathway that does not exist in *A. nidulans*. Ime2 homologs have been shown to regulate a number of pathways that do not involve Ndt80-like transcription factors (Irniger 2011).

XprG plays a major role in the response to carbon starvation in *A. nidulans* (Katz *et al.* 2013). We have previously proposed that the common feature of Ndt80-like proteins is a role in nutrient sensing, and this may be the original role for this group of transcriptional activators (Katz *et al.* 2006, 2013). Is ImeB responsible for modulating XprG levels in response to nutrient stress? The *imeBΔ* mutant has increased levels of extracellular protease, particularly in response to nitrogen starvation, and increased levels of the *xprG* transcript in response to carbon starvation. However, ImeB does not appear to play a role in autolysis or the induction of *chiB* in response to carbon nutrient stress. Mutations in *imeB* do not increase extracellular protease production or *xprG* and *ndtA* expression in the absence of nutrient stress, so if ImeB is indeed involved in nutrient signaling it cannot act alone. Another protein must block transcription of these genes when nutrients are present. As previous studies (Katz *et al.* 2008) have shown that the CreA DNA-binding protein, which mediates carbon catabolite repression, may modulate XprG activity, it is a likely candidate.

In addition to ImeB, a number of other negative regulators of XprG have been identified. Genetic evidence suggests that the AtmA kinase modulates XprG activity (Krohn *et al.* 2014) and the hexokinase-like proteins HxkC and HxkD regulate XprG activity or expression (Bernardo *et al.* 2007; Katz *et al.* 2000).

In contrast to *S. cerevisiae* and *N. crassa*, sexual development in *A. nidulans* is not triggered by nutrient limitation and requires nutrient-sufficient conditions (Dyer and O’Gorman 2012). Yet, we have shown that mRNA levels for the *ppgA* sex pheromone gene and *ndtA* regulator of sexual reproduction are elevated in response to nutrient limitation. Thus, it appears that NdtA still retains the capacity to respond to nutrient stress. Whether this has any biological relevance is unknown. The *imeBΔ* mutation leads to increased *ppgA* transcript levels in nutrient-sufficient conditions. This result is consistent with the observation that, in *N. crassa*, the *Δime-2* mutant produces abundant female sexual structures in nutrient-sufficient conditions that would normally repress sexual development (Hutchison and Glass 2010).

We have shown that ImeB is a negative regulator of *xprG* expression. Two observations suggest that ImeB may also regulate *ndtA* expression. 1) In the absence of nutrient stress, the *xprGΔ* mutation has no effect on *ppgA* expression. However, expression of the *ppgA* gene was increased in the *imeBΔ* mutant and this increase was NdtA-dependent. 2) A higher level of *ndtA* mRNA was detected in the *imeBΔ* mutant, though the level was not outside the 95% confidence interval of the control strain. As there is a higher level of the *xprG* transcript in the *imeBΔ* mutant, and XprG is a negative regulator of *ndtA* expression, we might expect to see a decrease in *ndtA* expression in an *imeBΔ* mutant rather than an increase if ImeB exerts no direct control over *ndtA* expression (*i.e.*, acts only through XprG).

It has been reported that ImeB is required for inhibition of sexual development by light but that no defect was observed in *A. nidulans* strains carrying the *veA1* mutation (Bayram *et al.* 2009). We have demonstrated that the *imeBΔ* mutation affects extracellular protease secretion, mycotoxin production, and transcript levels of the *ppgA*, *ndtA* and *xprG* genes in strains carrying the *veA1* mutation. Therefore, ImeB must have some functions that are VeA-dependent and some that are VeA-independent.

Within the ascomycetes, there are two groups of Ndt80-like proteins, those that are similar to *S. cerevisiae* Ndt80, and those that are similar to XprG and VIB-1 (Hutchison and Glass 2010; Katz *et al.* 2013) (Figure S3; Larkin *et al.* 2007). It is clear from studies in *A. nidulans* and *N. crassa* that the proteins in the two groups have different functions (Hutchison *et al.* 2012; Hutchison and Glass 2010; Katz *et al.* 2013). In some fungal species, the number of Ndt80-like transcription factors has expanded (*e.g.*, in *F. oxysporum*, *R. delemar*) and in others it has been reduced (*e.g.*, in *S. cerevisiae*) or eliminated (*e.g.*, in *S. pombe* and many basidiomycetes). Although, in most cases the function of the Ndt80-like proteins is not known, in the case of *C. albicans*, it is clear that expansion has been accompanied by the acquisition of new functions. The function of the *C. albicans* protein that is most closely related to *S. cerevisiae* meiosis-specific transcription factor Ndt80 (Q5A6P1, Figure S3) has not been reported. CaNdt80, which regulates genes involved in ergosterol biosynthesis, cell separation and hyphal development among many others, belongs to a novel Ndt80-like protein found only in the CTG clade of Saccharomycotina (Sellam *et al.* 2009, 2010). As CaNdt80 is required for virulence in *C. albicans*, it has been suggested that the gene duplication event which gave rise to CaNdt80 led to the ability of a number of fungi in the CTG clade to colonize mammalian hosts (Sellam *et al.* 2010). We have shown here that this extreme diversity in Ndt80-like proteins extends to the regulation of the genes encoding these proteins by Ime2 homologs and the ability of Ndt80-like proteins to regulate their own synthesis. Thus, the Ime2/Ndt80 signaling pathways display great flexibility in adapting to the lifestyle requirements of each species.

## Supplementary Material

Supporting Information
