# Supplementary material for: Extreme Diversity in the Regulation of Ndt80-Like Transcription Factors in Fungi
Source: G3 (Bethesda). 2015 Oct 22;5(12):2783–92. doi: 10.1534/g3.115.021378 (PMC4683649; doi:10.1534/g3.115.021378)
Supplement: Supporting Information [file supp_5_12_2783__index.html]

Extreme Diversity in the Regulation of Ndt80-Like Transcription Factors in Fungi — Supporting Information 

# Extreme Diversity in the Regulation of Ndt80-Like Transcription Factors in Fungi

## Supporting Information for Katz and Cooper, 2015

**Files in this Data Supplement:**

- Figure S1 - Alignment of the amino acid sequence of *S. cerevisiae* Ime2 (SGD, http://www.yeastgenome.org/) with the sequences of A. nidulans ImeB, MpkC, PhoA and PhoB (AspGD, http://www.aspgd.org/). (.pdf, 42 KB)
- Figure S2 - Production of extracellular proteases on medium containing 1% skim milk as a sole nitrogen or carbon source in the protein kinase mutants. (.pdf, 162 KB)
- Figure S3 - Phylogenetic tree of Ndt80-like proteins from selected fungi, animals and a slime mold. (.pdf, 108 KB)
- Table S1 - Oligonucleotides used in qRT-PCR and screening mutants. (.pdf, 38 KB).
