# Supplementary material for: Extreme Diversity in the Regulation of Ndt80-Like Transcription Factors in Fungi
Source: G3 (Bethesda). 2015 Oct 22;5(12):2783–92. doi: 10.1534/g3.115.021378 (PMC4683649; doi:10.1534/g3.115.021378)
Supplement: Supporting Information [file supp_g3.115.021378_FigureS1.pdf]

```
                *        20         *        40         *        60         *        80         *       100
Ime2 : MVEKRSRQSSSS---------GSEFS---------VPPDVDNPPLSIPLKTLSDRYQLIEKLGAGSFGCVTLAKAQFPLSNILGKQHDIRGTLMDQPKNG :  82
ImeB : --------------------MTVCY------------EHPSRGLTPTSMATLSDRFELMKEVGDGSFGSVAVARVRTAGSNIARR--------------- :  53
MpkC : --------------------MAEFI----------------RSDILGTTFETTSRYANLQPVGLGTAGVVCSAYDLIS---------------------- :  42
PhoA : MTSQRPTSSSSLLDIVANLASPKSLLFNAHHTNPPPSLPSQRAPTMDKSQQPSSFQQLEKLGEGTYATVFKGRNRQT---------------------- :  78
PhoB : --------------------MPRYL------------AVPPRRPLPVPRDDEHLAY----------LRQVYKGRNCQT---------------------- :  36

                *       120         *       140         *       160         *       180         *       200
Ime2 : HQNYITKTQGVVAIKTMMTKLHTLQDYTRV-REIKFILAIPANDHLIQIFEVFIDSENYQLHIVMECMEQNLYQMMKHR-RRRVFSIPSLKSILSQILAG : 180
ImeB : --------GTLVAIKTMKKTFDSLAPCLEL-REVIFLRTLPPHPHLVPALDIFLDPLTRKLHIAMEYMDGNLYQLMKAR-DHKYLDGKHVKSILYQILCG : 143
MpkC : --------EQVVAIKKMMKPFHSTSVAKRTYREVKLLRHLR-HDNLINMSDIFISPLE-DVYLVTELLGTDLHRLLNGK----PLESKFAQYFTYQILRG : 128
PhoA : --------GELVALKEIHLDSEEGTPSTAI-REISLMKELK-HESIVSLYDV-IHTEN-KLMLVFEYMDKDLKKYMDTRGDRGQLDQATIKSFMHQLMSG : 166
PhoB : --------GEMVALKEIHLDSEEGTPSTAI-REISLMKELH-HDNILSLYDV-VHTEN-KLMLVFEYMDQDLKKYMDTHGNHGQLEPAIVKSFAFQLLRG : 124

                *       220         *       240         *       260         *       280         *       300
Ime2 : LKHIHEHNFFHRDLKPENILITPST-----QYFEKEYMNQIGYQDNYVIKLADFGLARHVENKNP-YTAYVSTRWYRSPEILLRSGYYSKPLDIWAFGCV : 274
ImeB : LDHIHAHHFFHRDIKPENILVSTSAPNDSTFSRYSNLVTPPSTPTTYTVKIADFGLARETHSKLP-YTTYVSTRWYRAPEVLLRAGEYSAPVDMWAVGAM : 242
MpkC : LKYIHSAGVIHRDLKPGNLLINENC----------------------DLKICDFGLAR---VQEPQMTGYVSTRYYRAPEIMLTWQRYGSKVDLWSVGCI : 203
PhoA : IAFCHDNRVLHRDLKPQNLLINKKG----------------------QLKLGDFGLARAFGIPVNTFSNEVVTLWYRAPDVLLGSRTYNTSIDIWSAGCI : 244
PhoB : IAFCHDNRILHRDLKPQNLLINSKG----------------------QLKLADFGLARAFGIPVNTFSNEVVTLWYRAPDVLLGSRTYNTTIDIWSIGCI : 202

                *       320         *       340         *       360         *       380         *       400
Ime2 : AVEVTVFRALFPGANEIDQIWKILEVLGTPIKRSDFVNTNHITAPPPGGFWDDASNL-VHKLNLKLPYVEGSSLDHLLSSSQ-LSDLSEVVKKCLRWDPN : 372
ImeB : AVEIATLKPLFPGGNEVDQVWRVCEIMGSPGNWY-----SKSGAKLGGGEWKDGSRL-AQKLGFTFPKMAPHSMESILPAPQWPAALSNFVTWCLMWDPK : 336
MpkC : LAEMLLGRPLFPGTDHINQFWLITDLLGNPPDEV----------------IDRITTNNTRRVVKSMAKRNPRPLKEILPAAE-DAAL-NLLDNLLVFDPD : 285
PhoA : MAELYTGRPLFPGTTNEDQLQKIFRLMGTPSERS----------------WPGISQLPEYRAN--FHVYATQDLGLILPQID-PLGL-DLLNRMLQLRPE : 324
PhoB : IAEMFTGRALFPGTTNEDQLQKIFRVMGTPSERT----------------WPGVSQFPEYKSD--FPVYPPQDLRQVVPRID-PYGL-DLLRCMLRLQPD : 282

                *       420         *       440         *       460         *       480         *       500
Ime2 : ERATAQELCEMPFFENTVAS-------------------------------------------------------------------------------- : 392
ImeB : NRPTSSQAMQHEYFADAVDPLSVRPRSSTARLLGRKQSEKSVKSPTKDTYESPTLSSKPSWFRRSLIGRSESPALVEPEQPSKPTLPALDSNVDPQLLKP : 436
MpkC : RRISAEQGLMHPWMAPYHDP-------------------------------------------------------------------------------- : 305
PhoA : MRIDAHGALQHPWFHDLPQL-------------------------------------------------------------------------------- : 344
PhoB : LRISAVDALRHPWFNDPVSD-------------------------------------------------------------------------------- : 302

                *       520         *       540         *       560         *       580         *       600
Ime2 : --------------------------------------------------------------QVDARGNVTNTEQALIFAGINPVATNT---KPIYFNSS : 427
ImeB : KHSTSKRATWAHGAPMPILPSIRPVSPLSNAVTAQANPPAQESNTNAAKSSKKIGRQLSVNSHGNHYGDVHRQEAERALNGLGNNSTSTISQKESFFSHL : 536
MpkC : ---------------------------------------------------------------------------------------------------- :   -
PhoA : ---------------------------------------------------------------------------------------------------- :   -
PhoB : ---------------------------------------------------------------------------------------------------- :   -

                *       620         *       640         *       660         *       680         *       700
Ime2 : TKLPAETESNDIDISNNDHDSHAMCSPTLNQEKLTLVEFLNEFVEEDNDDHSIPD------------------VGTDSTISDSIDETELSKEIRNNLALC : 509
ImeB : RKRARRLSGRNQNAMNDDIEANAGCMPWSNRSSLALDSVNVSEAKQNSDFSELDKAVQNVRYSMDSAALGNVPVSITTPVEASKRQSMPQGSIRSMGDSP : 636
MpkC : ---------------TDEPVATEQFDWSFNDADLPLDTWKIMIYSEVLDFFQLTT------------------------------NAEPSGE-------- : 352
PhoA : ---------------------------------------------------------------------------------------------------- :   -
PhoB : ---------------------------------LPIDH-------------------------------------------------------------- : 307

                *       720         *       740         *       760         *       780         *       800
Ime2 : QLPDEEVLDHSLSNIRQLTNDIEIINKDEADNMEQLFFDLEIPEKDEFQRKQPFNEHADIDEDIVLPYVNNSNYTHTDRSHHRGDNVLGDASLGDSFNSM : 609
ImeB : ASMNGNGGPISSRTRRAMQMTNHPVHRYETPEEEDELLDEVLHSASSAAMRLAQAQMSDTSSNYSRPLCNEQSHNLPSPYPTPSPSAKCDGVSFGNEDAA : 736
MpkC : -------------------------------------------------------------------QSQNQSQSQSQAFTSSQDLQLASMLNLGEGELL : 385
PhoA : ------------------------------------------------------------------------------------QAQLQQQQMAGYGGMM : 360
PhoB : ----------------------------------------------------------------------------------------------GMEALT : 313

                *       820         *       840
Ime2 : PDFTPRNFLIPTLKKSREKFEPHLSNSNQHFGNVTF--------- : 645
ImeB : PNRRLPLSDEKSATSATRQWPTPPYDDGDWMNPASTKFLTGSTYR : 781
MpkC : PDFA-------ATIDPNKFGSVDYLMDGQSLDPNSFS-------- : 415
PhoA : P--------------PQQAY------------------------- : 366
PhoB : --------------------------------------------- :   -
```

Figure S1 Alignment of the amino acid sequence of *S. cerevisiae* Ime2 (SGD, http://www.yeastgenome.org/) with the sequences of *A. nidulans* ImeB, MpkC, PhoA and PhoB (AspGD, http://www.aspgd.org/). Amino acids that are conserved in all five proteins are shaded in black, in at least four of the proteins in dark grey and in at least two of the proteins in light grey. The alignment was generated with the MUSCLE (Edgar 2004) and the GeneDoc (Nicholas *et al.* 1997) computer programs.
