# Supplementary material for: Extreme Diversity in the Regulation of Ndt80-Like Transcription Factors in Fungi
Source: G3 (Bethesda). 2015 Oct 22;5(12):2783–92. doi: 10.1534/g3.115.021378 (PMC4683649; doi:10.1534/g3.115.021378)
Supplement: Supporting Information [file supp_g3.115.021378_FigureS2.pdf]

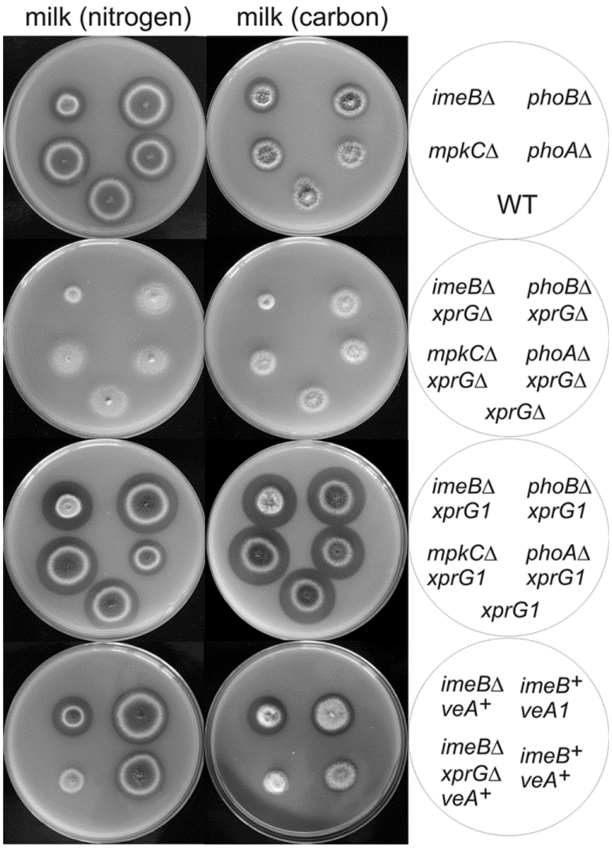


**Figure S2** Production of extracellular proteases on medium containing 1% skim milk as a sole nitrogen or carbon source in the protein kinase mutants. The clear halo surrounding the colonies is due to extracellular protease activity. The relevant genotype of the strains is shown. The full genotypes for the WT (MH2), *imeBΔ* (MK598), *mpkCΔ* (MK609), *phoAΔ* (MK552), *phoBΔ* (607), *imeBΔ xprGΔ* (MK601), *mpkCΔ xprGΔ* (MK608), *phoAΔ xprGΔ* (MK578), *phoBΔ xprGΔ* (604), *xprGΔ* (MK422), *imeBΔ xprG1* (MK582), *mpkCΔ xprG1* (MK612), *phoAΔ xprG1* (MK577), *phoBΔ xprG1* (606), *xprG1* (MK85), *imeB$^+$veA1* (MH2), *imeB$^+$veA$^+$* (MK562), *imeBΔ veA$^+$* (MK645) and *imeBΔ xprGΔ veA$^+$* (MK643) strains are given in Table 1
