# Supplementary material for: Extreme Diversity in the Regulation of Ndt80-Like Transcription Factors in Fungi
Source: G3 (Bethesda). 2015 Oct 22;5(12):2783–92. doi: 10.1534/g3.115.021378 (PMC4683649; doi:10.1534/g3.115.021378)
Supplement: Supporting Information [file supp_g3.115.021378_FigureS3.pdf]

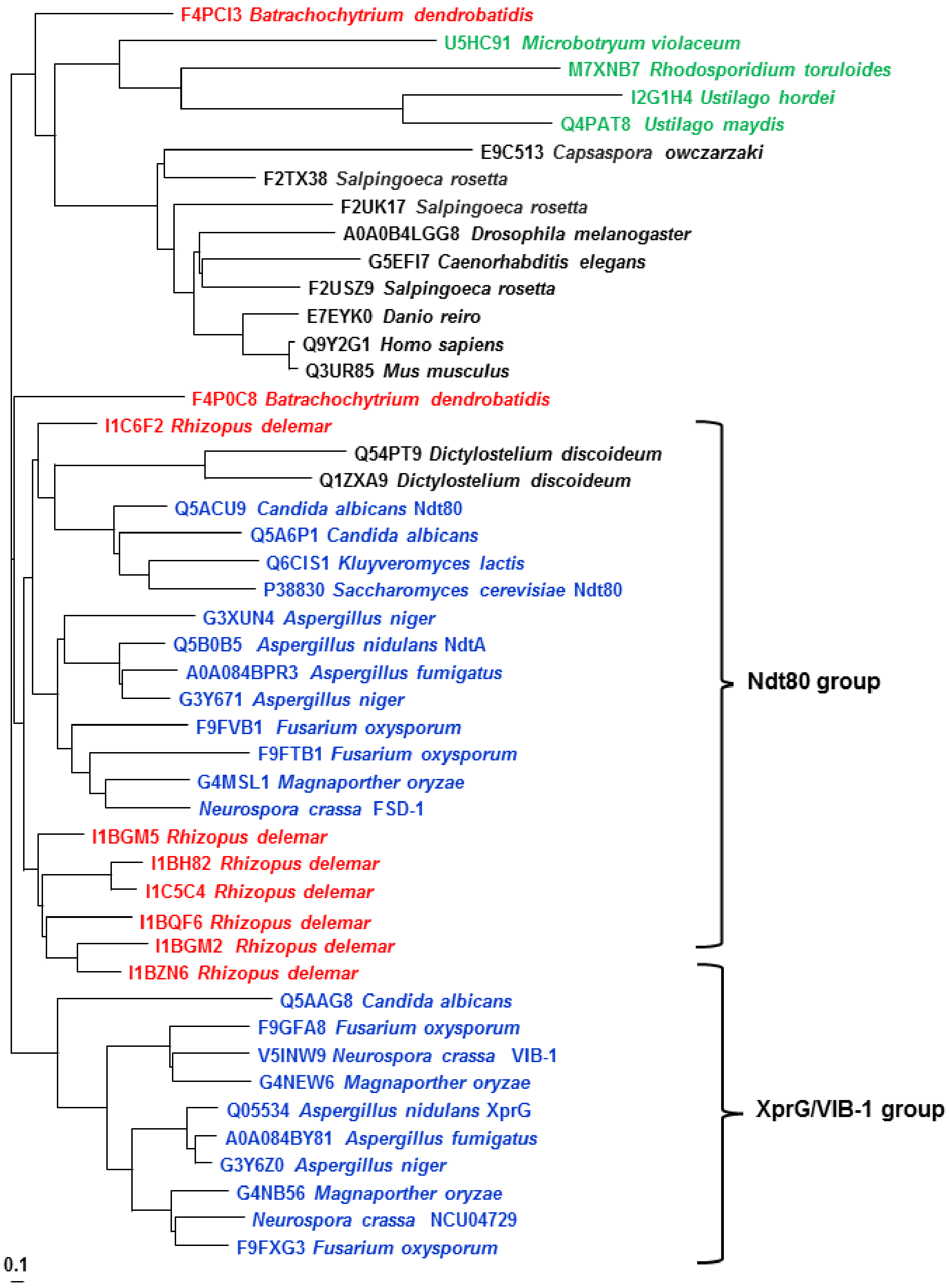

Figure S3 Phylogenetic tree of Ndt80-like proteins from selected fungi, animals and a slime mold. Sequences from ascomycete fungi are in blue, basidiomycete 41

fungi in green and other fungi in red. All sequences, with the exception of those from *N. crassa* are from the InterPro database (www.ebi.ac.uk/interpro/entry/IPR024061/taxonomy) and the accession numbers are given. The *N. crassa* sequences are from the *Neurospora crassa* database (www.broadinstitute.org/annotation/genome/neurospora/MultiHome.html). The alignment was generated with the MUSCLE (Edgar 2004) and ClustalW Phylogeny (Larkin *et al.* 2007) computer programs.
