# Supplementary material for: Extreme Diversity in the Regulation of Ndt80-Like Transcription Factors in Fungi
Source: G3 (Bethesda). 2015 Oct 22;5(12):2783–92. doi: 10.1534/g3.115.021378 (PMC4683649; doi:10.1534/g3.115.021378)
Supplement: Supporting Information [file supp_g3.115.021378_TableS1.pdf]

Table S1 Oligonucleotides used in qRT-PCR and screening mutants.

Gene	Primer name	Oligonucleotide sequence[a]	Position[b]	Purpose
actA	MK415	5'-AGAGGAAGTTGCTGCTCTCG-3' (F)	6	qRT-PCR
	MK416	5'-GGATACCACGCTTGGACTGT-3' (R)	193	
chiB	MK421	5'-ACGATCAGCAGGCTCAGAAC-3' (F)	425	qRT-PCR
	MK422	5'-TCTCCTGTAGCCGGAGCTTA-3' (R)	568	
ndtA	MK444	5'-CTGGCATGAGTTTCGACAGA-3' (F)	65	qRT-PCR
	MK445	5'-TATCGCTGAGGGGTGTCAAT-3' (R)	262	
ppgA	MK427	5'-TGCCGCTGAATTACAACATC-3' (F)	66	qRT-PCR
	MK428	5'-CGGAACCTGCACCATCTATT-3' (R)	212	
xprG	MK442	5'-GGAATATGCAGGGCACAGAT-3' (F)	56	qRT-PCR
	MK443	5'-TGGAGATAGATGGGCAAAGG-3' (R)	180	
phoA	MK375	5'-TACTTCCATGAGTTCGCGAC-3' (F)	-491	Detection of
pyroA^{Af}	MK323	5'-GATGGTCTCGAACTGACCTT-3' (R)	-474	*phoA::pyroA^{Af}*
phoB	MK434	5'-GCTTTAGCTTCACCTTCAGC-3' (F)	-328	Detection of
pyrG^{Af}	MK431	5'-CACCGTAAGTCAATTGCGAC-3' (R)	31	*phoB::pyrG^{Af}*
mpkC	MK432	5'-CTGGCATTGTAATCCGCCAA-3' (F)	-1111	Detection of
pyrG^{Af}	MK431	5'-CACCGTAAGTCAATTGCGAC-3' (R)	31	*mpkC::pyrG^{Af}*

[a]The sequences labeled (R) are complementary to the sense strand.

[b]The position of the 5' end of each primer in the coding region of the gene (excluding introns) is given. The sequences were obtained from the *Aspergillus* Genome Database (http://www.aspgd.org/).
